# Nanoparticle-Mediated Cavitation via CO_2_ Laser Impacting on Water: Concentration Effect, Temperature Visualization, and Core-Shell Structures

**DOI:** 10.1038/s41598-019-54531-1

**Published:** 2019-12-04

**Authors:** Man Hu, Feng Wang, Peng Huo, Xueqin Pan, Steven G. Johnson, Yoel Fink, Daosheng Deng

**Affiliations:** 1 Department of Aeronautics and Astronautics, Fudan University, Shanghai, 200433 China; 2 Department of Mathematics, Massachusetts Institute of Technology, Cambridge, MA 02139 USA; 3 Department of Materials Science and Engineering, Massachusetts Institute of Technology, Cambridge, MA 02139 USA; 4 Research Laboratory of Electronics, Massachusetts Institute of Technology, Cambridge, MA 02139 USA

**Keywords:** Applied physics, Fluid dynamics, Optical physics, Nanoparticles, Applied optics, Lasers, LEDs and light sources

## Abstract

By taking advantage of seeded polymer nanoparticles and strong photo energy absorption, we report CO_2_ laser impacting on water to produce cavitation at the air/water interface. Using a high-speed camera, three regimes (no cavitation, cavitation, and pseudo-cavitation) are identified within a broad range of nanoparticles concentration and size. The underlying correlation among cavitation, nanoparticles and temperature is revealed by the direct observation of spatiotemporal evolution of temperature using a thermal cameral. These findings indicate that nanoparticles not only act as preexisted nuclei to promote nucleation for cavitation, but also likely affect temperature to change the nucleation rate as well. Moreover, by exploiting a compound hexane/water interface, a novel core-shell cavitation is demonstrated. This approach might be utilized to attain and control cavitations by choosing nanoparticles and designing interfaces while operating at a lower laser intensity, for versatile technological applications in material science and medical surgery.

## Introduction

Since the seminal work by Lord Rayleigh in 1917^1^, cavitation has been extensively studied in various forms, such as flow cavitation, acoustic cavitation, and laser-induced cavitation^2–4^, offering diverse applications in microfluidic manipulation^5^, surface cleaning^6,7^, nanomaterials synthesis^8,9^, and medical treatment^10^. In particular, lasers impacting on liquids, ranging from a single droplet^11,12^ to soft biological tissues^13^, can generate vapor explosions and cavitation. Due to the intense laser-tissue interaction, lasers offer the potential to precisely manipulate or locally destroy biological tissues for many clinical applications including lithotripsy, tumor removal and neurosurgery^13–15^. Specifically, an ultrafast infrared laser can be selectively absorbed by water in the tissue to generate a rapid ablation or cutting process, holding promise for minimally invasive surgery down to the single-cell level^16^.

Generally, laser-induced cavitation is produced either by cascading ionization through focusing of high intensity short-pulsed lasers into transparent liquids (optical cavitation), or by direct heating due to strong absorption through irradiating long-exposure or continuous-wave lasers in highly absorbing liquids (thermocavitation)^17,18^. Recently, this cavitation has been manipulated into complex geometries while being encapsulated inside microchannels of PDMS devices^19^.

Moreover, cavitation can be controlled by seeded nanoparticles (NPs) immersed in water^20,21^. For example, in acoustic cavitation, polymer NPs (polyethylene) serve as nuclei to promote heterogeneous nucleation, while SiO_2_ NPs form stable bonds with water molecules to suppress cavitation. Metallic NPs (gold or aluminum) under incident light cause local boiling due to plasmonic resonance, generating vapor cavitation for solar energy harvesting^22–25^.

In this paper, by taking advantage of seeded polymer NPs and the strong photo energy absorption of water at the infrared wavelength, we report a CO_2_ laser impacting on water to produce cavitation at the air/water interface. Three regimes (no cavitation, cavitation, and pseudo-cavitation) are identified within a broad range of NP concentration and size. This cavitation allows the direct observation of spatiotemporal evolution of temperature, revealing the underlying correlation between cavitation and temperature. Moreover, by exploiting a compound hexane/water interface, a novel core-shell cavitation is demonstrated.

## Results

### Nanoparticle effect

#### Cavitation promoted by NPs

**Figure 1:**
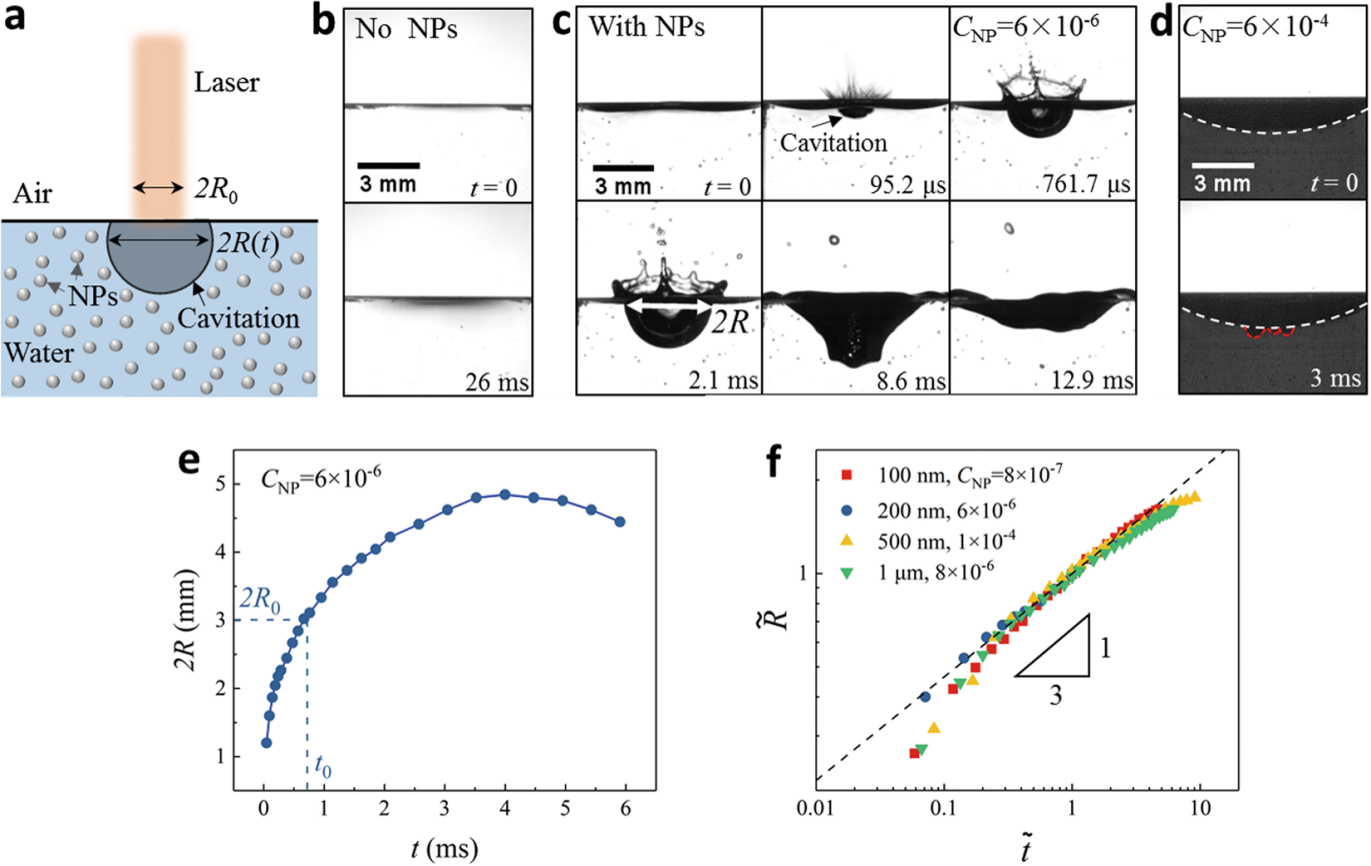
Observation of interfacial cavitation promoted by NPs. (**a**) Sketch of the produced cavitation during laser impacting on water. (**b**) No cavitation in distilled water (*P*_*laser*_ = 35 W, 2*R*_0_ = 3 mm). (**c**) High-speed images of cavitation with seeding NPs (*d*_*NP*_ = 200 nm and *C*_*NP*_ = 6 × 10^−6^). (**d**) Snapshots for pseudo-cavitation at higher concentration (*C*_*NP*_ = 6 × 10^−4^), the white line for the air/water interface and the red line for the indentation. (**e**) Dynamics of cavitation during the growth and collapse stage for *C*_*NP*_ = 6 × 10^−6^. (**f**) A power-law dependence of radius on time with an exponent *α* = 1/3 ($\tilde{R}\propto {\tilde{t}}^{\alpha }$, black line) for various *d*_*NP*_ and *C*_*NP*_.

Since water has a strong absorption peak of 832 cm^−1^ at the wavelength of 10.6 *μ*m^26^, resulting in a short penetration depth down to 12 *μ*m, the energy is dramatically attenuated when CO_2_ laser impacts on water (Fig. 1a). Arising from this strong photo-thermal effect, water is rapidly heated up to generate explosive vaporization^27,28^. As the laser intensity is about 5 × 10^2^ W/cm^2^, much lower than the typical ablation threshold (~10^4^ W/cm^2^), no cavitation but only vaporization is observed from high speed imaging (Phantom V611, Fig. 1b).

When the seeding polymer NPs (the diameter *d*_*NP*_ = 200 nm) are introduced into the distilled water at *C*_*NP*_ = 6 × 10^−6^ (the volume concentration, the ratio between the total NPs volume and water volume), cavitation is generated at the air/water interface at the same laser intensity in high-speed imaging [Fig. 1c and Supplementary Video (SV) 1]. At a much higher *C*_*NP*_ = 6 × 10^−4^ (Fig. 1d and SV 1), water becomes opaque, and no cavitation appears at the air/water interface (the white line). Instead, an unexpected indentation (the red line) is observed with a small size much less than the beam size, which is referred to as pseudo-cavitation.

#### Cavitation dynamics

At an NP concentration of *C*_*NP*_ = 6 × 10^−6^ (Fig. 1c), the interfacial cavitation with a hemi-spherical shape expands explosively within milliseconds, consistent with the capillary time scale ${\tau }_{0}={(\rho {R}_{0}^{3}/\gamma )}^{1/2}\approx 4\,{\rm{ms}}$, where *ρ* is the density of water, *γ* is the surface tension of water, and *R*_0_ is the radius of laser beam (2*R*_0_ = 3 mm). Once the radius (*R*) is increased beyond *R*_0_, the growth is gradually slowed down due to hydrostatic pressure, then cavitation is contracted quickly undergoing large deformations (the last two snapshots of Fig. 1c). The corresponding time-dependent radius *R*(*t*) is presented in Fig. 1e.

By a dimensional analysis $[\tilde{R}=R(t)/{R}_{0},\tilde{t}=t/{t}_{0},R({t}_{0})={R}_{0}]$, all the data during the cavitation growth for various *C*_*NP*_ and *d*_*NP*_ fall into a master curve, which is simply characterized by a power-law scaling $(\tilde{R}\propto {\tilde{t}}^{\alpha })$ with an exponent *α* = 1/3 over two orders of magnitude of $\tilde{t}$ (Fig. 1f). This scaling law likely implies a constant growth rate of cavitation volume (*dV*/*dt* = constant) as in the production limit^24^.

#### Phase diagram of cavitation

**Figure 2:**
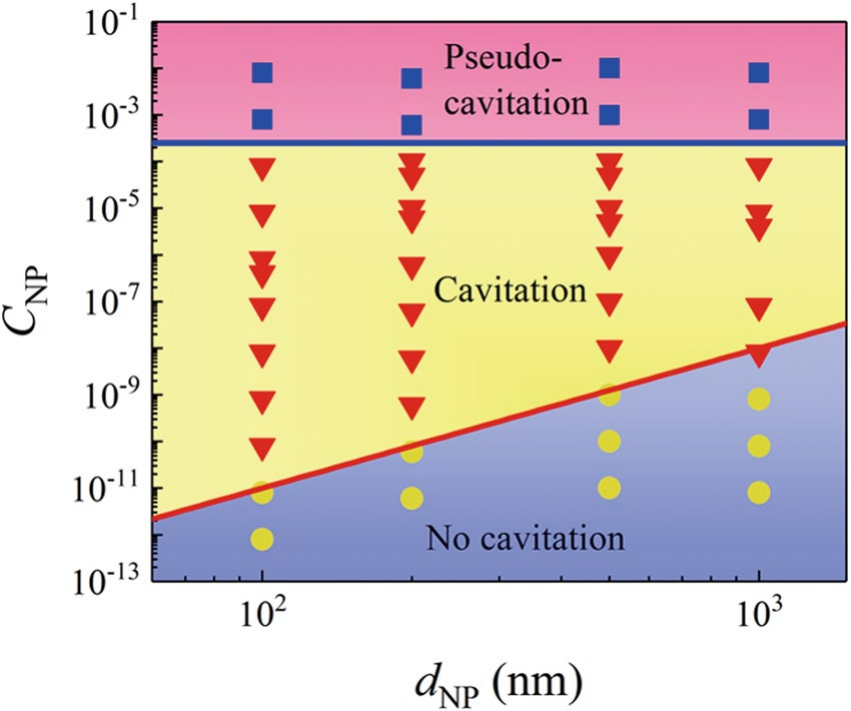
Phase diagram of cavitation dependent on *d*_*NP*_ and *C*_*NP*_. Three regimes of no cavitation, cavitation, and pseudo-cavitation are separated by two boundary lines. One is the red line plotted from Eq. (1) to show a threshold for cavitation formation at lower *C*_*NP*_, while the other is the blue line to indicate the threshold for pseudo-cavitation at higher *C*_*NP*_.

Now we proceed to establish the phase diagram of cavitation by impacting lasers on water samples within a broad range of *C*_*NP*_ up to ten orders of magnitude (10^−12^ to 10^−2^) and various *d*_*NP*_ (100, 200, 500 nm and 1 *μ*m) (Fig. 2). All the data are categorized into three groups: no cavitation (yellow circles) at lower concentration, cavitation (red triangles) at medium concentration, and pseudo-cavitation (blue squares) at higher concentrations.

These three groups are separated by two boundary lines: one is a low-concentration boundary for the transition from no cavitation to cavitation (the red line), while the other line is a high-concentration boundary for the transition from cavitation to pseudo-cavitation (the blue line). For the lower-concentration boundary, to overcome the interfacial energy to produce cavitation, at least a seeding NP is required to serve as pre-existing nucleus in water which is heated by the laser due to the strong absorption. Approximately, the volume of this heated water is ${V}_{heated}\sim {R}_{0}^{2}h$ (*h* for the penetration depth of CO_2_ laser in water). Then based on this simplified argument, the lower-concentration boundary is given by

1 $${C}_{NP}^{low}=\frac{{V}_{NP}}{{V}_{heated}}\sim \frac{{d}_{NP}^{3}}{{R}_{0}^{2}h}\sim {d}_{NP}^{3}.$$

${C}_{NP}^{low}\sim {d}_{NP}^{3}$ implies that the required *C*_*NP*_ for cavitation formation increases with *d*_*NP*_ with a power-law scaling of 3 (the red line), which is consistent with the observation. Once *C*_*NP*_ is above the low-concentration boundary, cavitation is formed.

However, as *C*_*NP*_ is further increased to be much higher, the pseudo-cavitation occurs and the corresponding high-concentration boundary is nearly independent of concentration, as indicated by the blue line. The exact mechanism of this transition is not fully understood, but possibly more NPs are likely aggregated at the cavitation interface to affect cavitation growth. In the limit that the cavitation surface is fully covered by NPs (similar to the Pickering emulsion stabilized by particles^29^ or the formed shell by particle accumulation in a Leidenfrost droplet^30^), the development or growth of cavitation can be prevented and only surface indentation occurs, leading to the transition from cavitation to pseudo-cavitation.

### Temperature visualization

#### Visualization of interfacial temperature

**Figure 3:**
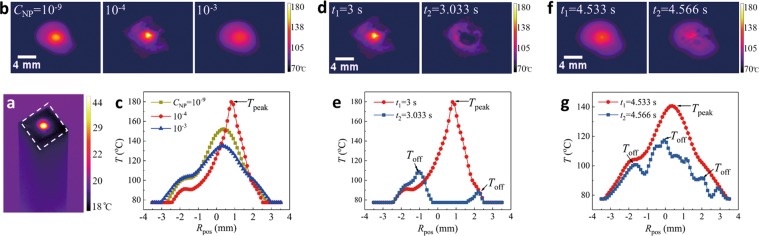
Visualization of interfacial temperature. (**a**) An overview for laser impacting on water, and the white rectangular indicating the air/water interface. (**b**,**c**) For three *C*_*NP*_ at 10^−9^, 10^−4^ and 10^−3^, the typical thermal images (**b**) and the temperature distribution dependent on the radial position (R_*pos*_) along a horizontal line through the center (**c**) at *t* = 3 sec (*t* = 0 sec refers to the moment of laser shotting on water surface). (**d**,**e**) At *C*_*NP*_ = 10^−4^ for cavitation, the typical thermal images (**d**) and the temperature distribution (**e**) before (*t* = 3 sec) and after (*t* = 3.033 sec) cavitation occurs. (**f**,**g**) At *C*_*NP*_ = 10^−3^ for pseudo-cavitation, the typical thermal images (**f**) and the temperature distribution (**g**) before (*t* = 4.533 sec) and after (*t* = 4.566 sec) pseudo-cavitation occurs. The thermal camera is operated at 30 Hz with the recorded temperature range from 80~200 °C. (*d*_*NP*_ = 500 nm, *P*_*laser*_ = 35 W).

Unlike the bulk cavitation immersed inside water^31–33^, interfacial cavitation is exposed to the ambient air, so the spatiotemporal evolution of interfacial temperature can be directly visualized using a thermal camera (FLIR A 6750sc, Fig. 3a and SV 2), revealing the correlation between cavitation and temperature. For the typical case of no cavitation, cavitation and pseudo-cavitation corresponding to *C*_*NP*_ = 10^−9^, 10^−4^ and 10^−3^, the thermal snapshots and temperature profiles are compared in Fig. 3b,c, after 3 seconds upon laser impacting on water (*d*_*NP*_ = 500 nm, *P*_*laser*_ = 35 W). The peak temperature (*T*_*peak*_), the highest temperature from the temperature profile of Fig. 3c is ~150 °C for no cavitation at *C*_*NP*_ = 10^−9^, then increases to ~108 °C at *C*_*NP*_ = 10^−4^, but drops down to ~130 °C at *C*_*NP*_ = 10^−3^.

Additionally, the temperature profiles are presented before the cavitation formation and right after the cavitation formation. At *C*_*NP*_ = 10^−4^ for cavitation (Fig. 3d,e), the water is in a superheated state and *T*_*peak*_ reaches as high as ~180 °C at *t* = 3 sec. Then after 33 ms (at *t* = 3.033 sec), the cavitation is generated, and temperature decreases in the center while off-center satellite peaks (*T*_*off*_) occur locally away from the center. But at higher *C*_*NP*_ = 10^−3^ for pseudo-cavitation (Fig. 3f,g), *T*_*peak*_ is ~140 °C. After 33 ms, the pseudo-cavitation is generated with many local off-center satellite temperature peaks, as shown in Fig. 3g.

The radial symmetry of the temperature profile is preserved for no cavitation at the lower *C*_*NP*_ = 10^−9^, but is broken with the appearance of off-center *T*_*off*_ at a higher concentration for either cavitation or pseudo-cavitation (Fig. 3 and SV 2). The off-center *T*_*off*_ might be caused by the strong convection to push the central region of heated liquid outward radically associated with cavitation or pseudo-cavitation. Indeed this dominant convection is correspondingly correlated with a large Péclet Number, $Pe=Lu/\alpha \sim {10}^{3}\gg 1$ ^34^, where *α* ~ 0.6 w/(m · k) is thermal diffusivity of water, *l* ~ mm is the characterized length scale, and *u* ~ m/s is typical flow velocity.

#### Surface temperature dependent on *C*_*NP*_

**Figure 4:**
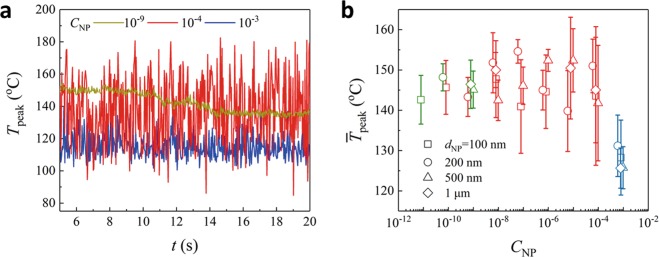
Temperature dependent on NPs. (**a**) The evolution of peak temperature (*T*_*peak*_) for *d*_*NP*_ = 500 nm with *C*_*NP*_ at 10^−9^, 10^−4^ and 10^−3^. (**b**) Time-averaged peak temperature $({\bar{T}}_{peak})$ is related with both *C*_*NP*_ and *d*_*NP*_, resulting in no cavitation (green symbols), cavitation (red symbols) and pseudo-cavitation (blue symbols). (*P*_*laser*_ = 35 W).

Here, the evolution of *T*_*peak*_ is presented at *C*_*NP*_ = 10^−9^, 10^−4^ and 10^−3^ (Fig. 4a). At a lower *C*_*NP*_ = 10^−9^ for no cavitation, the water temperature is elevated arising from the strong absorption of laser energy by water, and *T*_*peak*_ is relatively stable around 150 °C. However, at a higher *C*_*NP*_ = 10^−4^ for cavitation, the surface temperature presents strong fluctuations and can reach as high as 180 °C, which is clearly larger than the temperatures recorded for *C*_*NP*_ = 10^−9^. Besides the water absorption, polymer NPs here might also be heated up by the laser, and this additional absorbed energy is localized due to the small thermal conductivity of polymer NPs, resulting in an further increased temperature. From the classical nucleation theory for cavitation formation which is strongly dependent on temperature, the nucleation rate grows by orders of magnitude when the water temperature is increased by 30 °C. But at a much higher *C*_*NP*_ = 10^−3^ for the pseudo-cavitation, the recorded temperature drops down to ~100 °C with much weaker fluctuations. Rayleigh scattering from NPs (*d*/*λ* ≈ 1/20) might play a key role to cause the dramatic reduction of light absorption by water, leading to the sudden drop of the measured temperature. The plasmonic effect associated with metal NPs, by which the liquid temperature is increased with NPs concentration, should be irrelevant here for polymer NPs.

#### Correlation among cavitation, temperature and NPs

The whole process of cavitation at *C*_*NP*_ = 10^−4^ with strong fluctuations of *T*_*peak*_ (the red line) can be comprehended as follows: Initially, water is superheated through strong absorption upon the arrival of laser. Then, cavitation is produced by the heterogeneous nucleation at the elevated temperature. Subsequently, an explosion is followed by creating a plume and splashing of tiny droplets (Fig. 1c), which may temporarily reduce the laser intensity at the water interface and also expose colder water. Eventually, the temperature of the liquid is reduced and the next cycle of heating begins. Consequently, the growth and collapse of the cavitation leads to the strong fluctuations of temperature.

Cavitation associated with NPs and temperature is identified (Fig. 4b) for various *d*_*NP*_ with a broad range of *c*_*NP*_ [no cavitation (green symbols), cavitation (red symbols), and pseudo-cavitation (blue symbols)]. ${\bar{T}}_{peak}$ (the time-averaged *T*_*peak*_) for no cavitation and cavitation is similar, which is attributed to the photothermal effect of water with strong absorption at the infrared wavelength. However, cavitation (red symbols) is characterized by strong fluctuations (larger error bar), as the heating and cooling process is associated with cavitation. At a much higher *C*_*NP*_ = 10^−3^ for pseudo-cavitation, ${\bar{T}}_{peak}$ drops dramatically, which might be due to Rayleigh scattering from NPs. Hence, polymer NPs not only act as preexisted nuclei to promote nucleation for cavitation, but also likely affect temperature to change the nucleation rate.

### Application to fabrication of core-shell cavitation

#### Cavitation at the air/hexane interface

**Figure 5:**
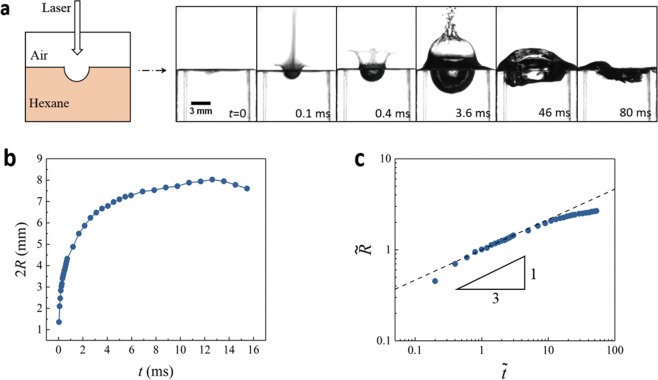
CO_2_ laser impacting on hexane. (**a**) High-speed images of cavitation formed in hexane. (**b**) Dynamics of cavitation in hexane. (**c**) A power-law dependence of radius on time with an exponent *α* = 1/3 ($\tilde{R}\propto {\tilde{t}}^{\alpha }$, black line).

Besides water, cavitation also can be generated by lasers impacting on other liquids. For example, we studied a CO_2_ laser impacting on hexane (Table 1), and high-speed imaging shows the cavitation dynamics (Fig. 5a,b). During the growth stage (Fig. 5c), a power-law scaling $(\tilde{R}\propto {\tilde{t}}^{\alpha })$ with an exponent *α* = 1/3 still holds in the production limit^24^.

**Table 1:** Properties of water and hexane.

	Density (g · cm^3^)	Boiling point (°C)	Latent heat (kJ · kg^−1^)	Viscosity (mPa · s)	Absorption coefficient at 10.6 *μ*m (cm^−1^)	Absorption depth (*μ*m)
Water	1.0	100	2260.4	1.002	832	12
Hexane	0.66	68	384.3	0.3	8	1250

#### Core-shell cavitation at the water/hexane interface

**Figure 6:**
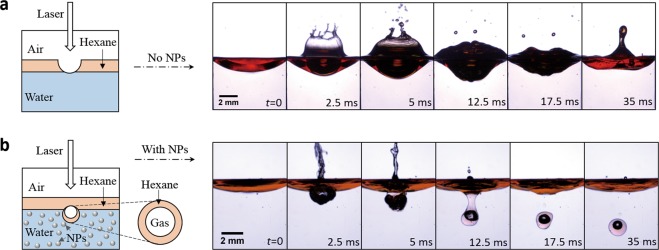
Application to fabricate core-shell cavitation. (**a**) Only cavitation formed in the upper hexane layer when NPs are absented in water. (**b**) A core-shell cavitation is observed when NPs are seeded in water (*d*_*NP*_ = 500 nm, *C*_*NP*_ = 10^−5^ and *P*_*laser*_ = 35 W).

Here, by decorating a thin layer of hexane on top of water (Fig. 6a,b), we further demonstrate a core-shell cavitation by impacting a CO_2_ laser on this compound fluid interface. The thickness of top liquid hexane is designed to be on the order of the penetration depth, around 1 mm (an absorption peak of 8 cm^−1^ at 10.6 *μ*m, Table 1), so that the laser remains strong enough to impact on the compound water/hexane interface, likely resulting in an interesting cavitation.

For distilled water without NPs (Fig. 6a), cavitation is observed in the upper layer of hexane with dye (see Methods), while no cavitation is formed in water. In contrast, for water with seeding NPs (*d*_*NP*_ = 500 nm and *C*_*NP*_ = 10^−5^) (Fig. 6b), interestingly a core-shell cavitation is obtained. Further, this core-shell cavitation is observed at various *d*_*NP*_ and *C*_*NP*_. The gas component inside the inner core might be composed of hexane vapor, water vapor, or their mixture, which is produced at the elevated temperature and encapsulated by the hexane. This core-shell cavitation demonstrates the controllable capability to fabricate novel cavitations by designing the compound interface.

## Discussion and Outlook

For bulk cavitation inside water, the pressure in the cavitation can be estimated by an isothermal or adiabatical approximation, and the associated dynamics can be described by a Rayleigh-Plesset equation. Here the produced cavitation at the air/water interface is exposed to the ambient air and the atmospheric pressure, and the strong photothermal process during the laser impact on water leads to a complicated heat transfer and temperature distribution. Hence, an accurate calculation of the pressure and temperature might be challenging theoretically to quantitatively characterize the dynamical process including both expansion and collapse for the system here.

By taking advantage of NPs and strong absorption of water, the laser intensity for thermal cavitation is reduced by 2 orders of magnitude (down to 10^2^ W/cm^2^), making possible the operation at a lower laser intensity. Typically, during a laser pulse (down to nanoseconds or picoseconds) impacting on liquids, an extremely high laser intensity (up to ~10^11^ W/cm^2^)^35^ triggers a rapid and complicated response from the liquid at an initial stage, resulting in plasma formation and shock waves^12,36^. But here the continuous laser with intensity down to 10^2^ W/cm^2^ is much lower than the optical breakdown threshold, and shock is less obvious and can be negligible. The lower laser intensity also results in a much lower temperature of cavitation (in the range of 110~160 °C), compared to the elevated temperature of cavitation (up to thousand degrees) in conventional optical breakdown^37,38^.

Additionally, more future work is required to thoroughly understand the complicated ${\bar{T}}_{peak}$ dependence on NP parameters such as *d*_*NP*_ and *C*_*NP*_. Yet from the experimental point of view, despite the limited frame rates of the thermal camera constraining the capability to capture the highest temperature during the fast process of cavitation, the direct visualization of the spatiotemporal evolution of temperature has evidently revealed the inherent correlation between the temperature, NPs and cavitation.

 A compound interface^39^ might be designed with various liquids to attain more sophisticated structured cavitation with promising functionalities, such as the separation and selection of liquid phases, or the delivery of targeted liquid. Although complicated liquid structures can be generated by other approaches such as a microcapillary device^40^, the structured cavitation here might be particularly applicable for laser-related technological applications, such as CO_2_-laser surgery^14,15^, and the precise manipulation of microstructures in materials science^41^. Moreover, the novel core-shell cavitation together with the recently reported conical interfaces^42^ might inspire more future studies on the compound interface of two immiscible fluids impacted by a laser beam and the subsequent diverse patterns and structures.

## Conclusion

In summary, by taking advantage of seeding NPs and a strong photothermal effect, we report a CO_2_ laser impacting on water to produce interfacial cavitation, allowing the direct visualization of the spatiotemporal evolution of temperature to reveal the underlying correlation among cavitation, nanoparticles and temperature. By systematically investigating a broad range of NPs concentration and size, a phase diagram is established with three identified regimes (no cavitation, cavitation, and pseudo-cavitation). These findings indicate that NPs not only act as preexisted nuclei to promote nucleation for cavitation, but also likely affect temperature to change the nucleation rate as well. Moreover, by exploiting a hexane/water interface, a core-shell cavitation is demonstrated. This work offers more opportunities to attain and control cavitations by choosing NPs and designing the compound interface, while operating at a lower laser intensity, for versatile technological applications in material science and medical surgery.

## Methods

### Experimental setup

**Figure 7:**
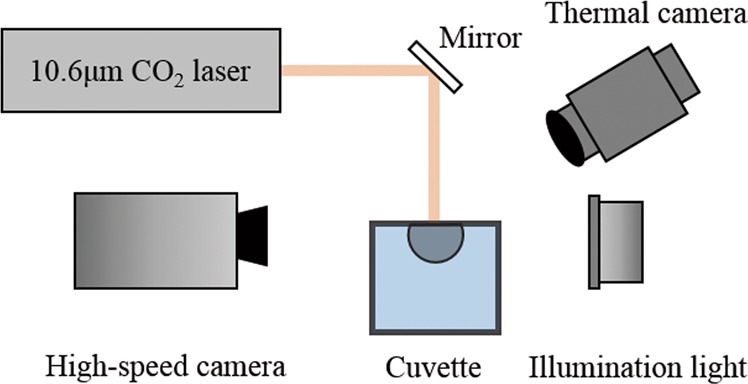
Experimental setup for laser impacting on water.

A schematic diagram depicting the experimental setup is shown in Fig. 7. A continuous-wave CO_2_ laser (Access laser, AL30D, the power *P*_*laser*_ = 35 W, the diameter of Gaussian beam *D*_0_ = 2*R*_0_ = 3 mm) with wavelength of 10.6 *μ*m is impacted on water in a glass cuvette (10 mm × 10 mm × 5 mm). The output laser beam is reflected by a lens and then irradiated downward into the glass cuvette. The collimated laser beam diameter is 3 mm, and the output laser power is 35 W. Cavitation is visualized using bright-field photography by a high-speed camera (Phantom V611 from Vision Research, up to 10^6^ frames per second) or a colored high-speed camera (Phantom C110), coupled with a macro lens (Tokina 100 mm F2.8d). A xenon lamp with a maximum power of 350 W used as an auxiliary lighting source is put on the other side of the cuvette, to project the cavitation’s shadow on the Phantom fast camera for better imaging effect. A thermal camera (FLIR A 6750sc) is adopted to acquire the temperature distribution on the water surface during the cavitation process.

For the seeding polymer NPs (ThermoFisher Scientific, polystyrene with an absorption peak about 170 cm^−1^ at the wavelength of 10.6 *μ*m^43^), the volume concentration (the ratio between the total NPs volume and the water volume) such as *C*_*NP*_ = 6 × 10^−3^, is obtained by diluting 1 ml polystyrene NPs (10^−2^ g/ml) with the distilled water (Shanghai Dienabiotech) into 10 ml solution in the volumetric flask. By the similar procedure, the various concentrations are obtained.

For the dye of hexane, since both water and hexane are transparent liquids, the hexane was saturated with the organosulfur (tetrathiafulvalene) into the orange color, and the compound interface is better visualized by a colored fast-speed camera (Phantom C110, SV 3).

## Supplementary information

Supplementary video 1

Supplementary video 2

Supplementary video 3
